# Assessing green production efficiency and spatial characteristics of China’s real estate industry based on the undesirable super-SBM model

**DOI:** 10.1038/s41598-024-67506-8

**Published:** 2024-07-16

**Authors:** Bo-Wen An, Pei-Yuan Xu, Chun-Yu Li, Lan-Yue Zhang, Qiu-Ping Guo

**Affiliations:** 1 College of Economics and Finance, Huaqiao University, Quanzhou, 362021 Fujian China; 2 Tianfu College, Southwestern University of Finance and Economics, Mianyang, 621050 Sichuan China; 3 School of Digital Economics, Sichuan University Jinjiang College, Meishan, 620860 Sichuan China; 4 Pan Asia Business School, Yunnan Normal University, Kunming, 650092 Yunnan China

**Keywords:** GPE of real estate industry, Spatial centroid evolution, Spatial clustering trend, Spatial disparity pattern, Distribution dynamic evolution, Energy and society, Environmental economics, Sustainability

## Abstract

As China strives to balance rapid urbanization with environmental conservation, increasing attention is being paid to the pursuit of green production efficiency (GPE) in the real estate industry. The undesirable super-SBM model was used to calculate the GPE of China's real estate industry from 2001 to 2020. Additionally, GPE spatial distribution characteristics in China's real estate industry were analyzed using the standard deviation ellipse (SDE), Moran’s index, Theil index, random kernel density estimation (RKDA), and spatial Markov chain (SMC) methods. The GPE exhibited a U-shaped trend, with 2008 as the inflection point, first decreasing and then increasing. It reached a maximum value of 0.747 in 2020. The Theil index increased from 0.043 to 0.121 nationwide, indicating the overall characteristics of low-level slow growth, and imbalance. Discrepancies in input–output scales, the southward shift of economic centers, and population movements contribute significantly to the disparities between the east and west, north and south, and regions divided by the Hu Huanyong Line (Hu Line). The GPE exhibited club convergence characteristics; however, polarization phenomena exist in local areas. Spatial spillover effects were also observed in GPE. Finally, we provide recommendations for promoting green development in the real estate industry, including green building technology, fiscal subsidy investment, and population migration management.

## Introduction

In recent years, sustainable development has become a focal point in various industries globally, with particular attention directed toward the real estate sector owing to its significant environmental footprint and pivotal role in economic growth^1–3^. In China, the world's largest real estate market, the pursuit of green production efficiency (GPE) has garnered increasing attention as the nation endeavors to balance rapid urbanization with environmental conservation. The importance of this topic stems from the pressing need to reconcile economic development with environmental preservation, particularly in countries experiencing rapid urbanization and industrialization, such as China^3–5^. As a significant contributor to resource consumption and pollution, the real estate industry has the potential to enhance green production practices and overall environmental sustainability.

The core issue addressed in this study is how the Chinese real estate industry can enhance its GPE. Specifically, we focused on how the real estate industry can effectively utilize resources, reduce environmental pollution, and achieve higher production efficiency. Guided by these issues, we investigate the current status of GPE in the real estate industry across different geographical spaces and explore their influencing factors, thereby revealing existing challenges and potential developmental opportunities.

Existing research has primarily focused separately on production efficiency and green development in the real estate industry, with few studies combining both aspects^6–9^. Most existing studies have primarily focused on traditional efficiency analysis methods, neglecting the impact of undesirable outputs, such as carbon dioxide emissions, thereby requiring a comprehensive assessment of actual production conditions. Therefore, the contribution of this study lies in introducing an undesirable super-SBM model that comprehensively considers undesirable outputs and inputs during the production process, enabling a more accurate evaluation of GPE in the real estate industry. Additionally, through spatial analysis methods, we explored regional differences in GPE and their influencing factors to provide scientific evidence for the formulation of targeted policies.

## Literature review

### Calculation of GPE in the real estate industry

GPE refers to maximizing output while ensuring economic benefits and minimizing resource consumption and environmental pollution^10,11^. The connotation of GPE in the real estate industry involves the unity of resource utilization, environmental protection, and economic benefits. From a resource utilization perspective, GPE requires real estate enterprises to minimize natural resource consumption during development, construction, and operational processes to achieve effective and renewable resource utilization^9,12^. GPE entails responsibilities and obligations for environmental protection, requiring real estate enterprises to focus on reducing environmental pollution, protecting the ecological environment, and achieving coordinated development between production and the environment for project planning and implementation^6–8^. GPE must consider economic benefits by enhancing green technology and management levels, reducing production costs, improving enterprise competitiveness, and maximizing economic benefits.

The measurement of GPE in the real estate industry necessitates consideration of the impact of production-related pollution on the environment, often referred to as the production efficiency of undesirable outputs^12,13^. Data envelopment analysis is extensively applied in the real estate industry owing to its flexibility in evaluating efficiency across multiple inputs and outputs without requiring a specific production function. Lins et al. used the CCR and BCC models to gauge production efficiency within the real estate sector^14^. However, these models must address input–output redundancies. Recognizing this limitation, Liu et al. adopted the SBM model^15^. Production-related undesirable outputs such as carbon dioxide emissions significantly affect the ecological environment. Consequently, An et al. chose the SBM model, which accounts for undesirable outputs, to measure GPE^12^. To mitigate instances in which efficiency values reach 1 and become incomparable using the method above, Guo et al. employed the super-SBM model to measure GPE more effectively^16^. The input and output variables typically include human capital, funds, and land. Human capital metrics include labor force and enterprise count; fund metrics involve investments in real estate development, fixed assets, and sales costs; and land metrics include construction areas, new construction, and land purchases^13–16^. Outputs encompass both desirable and undesirable aspects. Desirable outputs encompass housing construction, enterprise revenue, and social benefits, whereas undesirable outputs typically include pollutants such as sulfur dioxide, chemical oxygen demand, and carbon dioxide emissions^13,16,17^.

### Factors influencing GPE in the real estate industry

Technological factors play a crucial role in the real-estate industry’s GPE. Adoption of environmental protection technologies shape the ecological performance of real estate projects. Using energy-saving and environmentally friendly architectural design and construction technologies can reduce energy consumption and emissions, thereby enhancing GPE. Such technology application reduces the environmental footprint of projects and promotes sustainable development to a certain extent^6,18^. Building material selection has a significant impact on GPE. Choosing renewable, low-carbon, and environmentally friendly materials can effectively reduce resource consumption and environmental pollution, thereby improving the overall green performance of projects. The material selection decision is closely related to economic benefits and directly affects ecological environmental sustainability^7,8^. Efficient energy utilization is a critical factor affecting GPE. By introducing energy-saving equipment, intelligent control systems, and renewable energy technologies, it is possible to effectively improve the energy utilization efficiency, reduce energy consumption during the production process, and further promote the realization of green production. Applying such technologies not only enhances project competitiveness, but also lays a solid foundation for the industry's sustainable development^9,19^.

Economic factors play a significant role in the real estate industry’s GPE. Government policy formulation is crucial for improving GPE. Governments promote green development in the real estate industry by enacting environmental laws and regulations, establishing environmental standards, and providing financial and tax incentives. These policy tools enhance overall GPE. Government policy measures not only influence enterprise behavioral choices but also have far-reaching effects on environmental awareness and behaviors of the entire market, promoting green upgrading and transformation of economic structures^20,21^. Market mechanisms play an essential role in shaping GPE. The degree of market demand for environmental products and services, price levels, and the competitive landscape of the environmental industry directly affect the real estate industry’s GPE. Market mechanisms serve as a necessary means of resource allocation and drive the development of green technologies and environmental industries, which are profoundly significant for enhancing the overall industry's GPE^22,23^. Investor preference is a critical factor influencing GPE. The level of investor recognition of environmental projects, investment willingness, and degree of financial support directly impact the GPE of the real estate industry. Investor attitudes and behaviors reflect the market's confidence in and recognition of the green industry and influence enterprise green investment decisions and behavioral performance^24,25^.

Population factors play a crucial role in shaping real estate industry GPE. Evolution of the population structure directly influences changes in the demand structure and quality standards for real estate products, thus impacting GPE. With a continuously aging population, the emphasis on the ecological environment and the pursuit of high-quality living are gradually enhanced, driving the real estate industry toward greening and quality improvement, thereby enhancing GPE^26–28^. The increase in urbanization level affects GPE. Acceleration of the urbanization process leads to intensified resource consumption and environmental pollution, thereby prompting the transformation and upgrading of the real estate industry, which is positively significant for GPE improvement. Transformation of the economic structure and lifestyle brought about by urbanization provides favorable conditions for popularizing environmental awareness and applying green technology^22,29^. Enhancing resident environmental awareness is a critical factor affecting GPE. The degree of recognition of environmental products and services by residents, the extent of adoption of environmental behaviors, and conscious actions toward environmental protection directly influence the level of GPE in the real estate industry. This increase in awareness guides enterprises to actively promote green production and stimulate market demand for environmental products and services, thereby promoting the entire industry toward a more sustainable direction^25,30^.

### Spatial distribution of GPE in the real estate industry

Research on GPE spatial analysis can be categorized into three primary spatial partitioning types. One approach involved dividing the research into eastern, central, and western regions. Studies within these regions indicate a widening regional disparity in China’s green total factor productivity (GTFP), particularly prominent in the eastern region. This discrepancy is primarily attributed to the emphasis on industrial upgrading and technological innovation, which significantly impact GTFP^10,11,31^. Another set of studies focused on the economic imbalance between China's northern and southern regions. The findings suggest a 'fast south and slow north' phenomenon, attributed to slower capital accumulation in the north, coupled with an inadequate economic structure and labor force migration toward the south. Moreover, the 'increase in the south and decrease in the north' pattern in marketization contributes significantly to the widening wage disparity between these regions^32,33^. Additionally, analyses centered around the Hu Huanyong Line (Hu Line) revealed a southeastward shift due to population migration. However, developments in the Yellow River Basin offer prospects for breakthroughs in population migration toward the west^34–36^.

From a research methodology standpoint, studies of spatial characteristics in production activity often employ four primary methods. First, the Theil index, variance decomposition, and convergence models enabled an examination of spatial heterogeneity. This approach revealed pronounced spatial differentiation characteristics of GPE across China^37,38^. Second, geographic detector methods such as location entropy, Moran’s index, and Local Indicators of Spatial Association (LISA) agglomeration analysis have been instrumental in researching spatial agglomeration. These methods effectively describe spatial clustering tendencies in green industrial development^39,40^. The third method involved a combination of social network analysis and QAP regressions to scrutinize spatial correlations. This approach has contributed to confirming the existence of spatial network structures within China's population migration patterns and GPE dynamics^41,42^. Finally, the study of spatial evolution trends incorporated distribution dynamics methods, including random kernel density estimation (RKDE) and a spatial Markov chain (SMC). These methods facilitate depiction of the spatial structural evolution of China's green innovation capability^43,44^.

### Primary contributions

Compared with existing studies, this study introduces several significant innovations. First, it innovates the calculation methods used to assess GPE in the real estate industry. Employing the super-SBM model with undesirable outputs, this study considers input indicators such as labor force, capital, and land. Desirable outputs encompass physical, economic, and social aspects, whereas carbon dioxide emissions comprise undesirable outputs. Proxy variables were carefully selected for each indicator to mitigate endogeneity among indicators. Second, we introduce novel methods for studying the spatial characteristics of GPE in the real estate sector. By employing various approaches, such as the standard deviation ellipse (SDE), Moran’s index, Theil index, RKDE, and SMC, this study conducted a comprehensive analysis of GPE from both static and dynamic perspectives. This multifaceted approach enables the exploration of GPE spatial distribution patterns, disparity characteristics, and evolutionary dynamics, ensuring a comprehensive understanding without relying on singular research perspectives or limited conclusions. Third, this study innovates the spatial division scale applied to examine GPE in China's real estate industry. Recognizing the new patterns of China's economic development and the changing landscape of population migration, this study delves into GPE across three distinct geographical divisions: the eastern, central, and western regions; northern and southern regions; and eastern and western areas separated by the Hu Line. This multifaceted approach aims to uncover the underlying reasons for the spatial disparity patterns observed in GPE in China's real estate industry.

## Materials and methods

**Figure 1:**
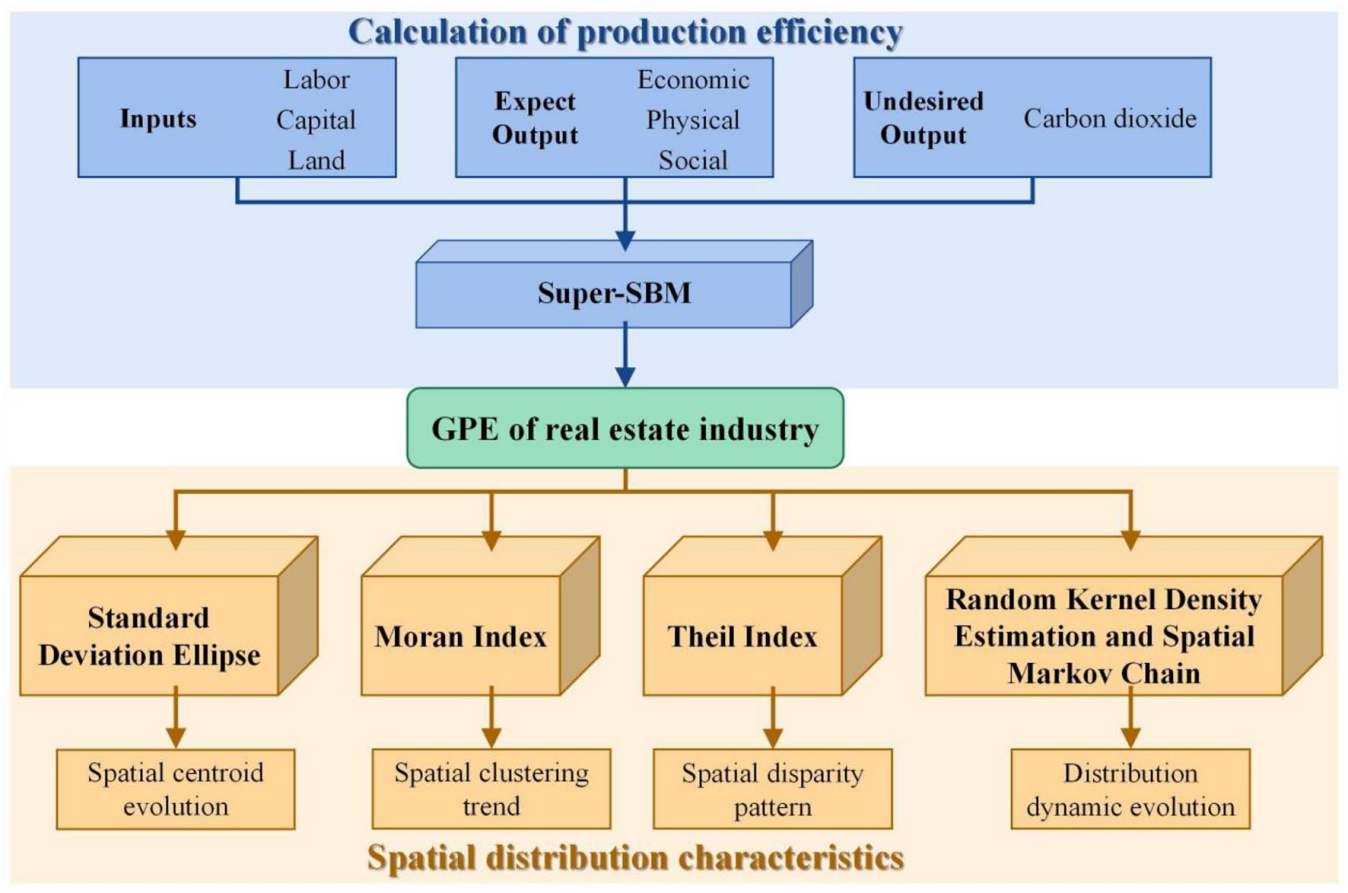
Research framework.

We proposed an integrated research framework to measure the GPE of China's real estate industry and explored its spatial distribution characteristics (Fig. 1). Starting from the definition of GPE in the real estate sector, we selected input–output indicators and used the super-SBM model for calculation. The spatial centroid evolution of GPE was analyzed using the SDE method. We examined the spatial clustering trend of GPE using Moran’s index, whereas we investigated the spatial disparity pattern using the Theil index. Finally, we analyzed GPE dynamic evolutionary distribution using RKDE and SMC techniques.

### Selection of variables and spatial division

Real estate green efficiency can be defined as maximizing economic output while minimizing the environmental impact of the production, utilization, and management of real estate resources. This concept entails the application of sustainable development principles and environmental technologies in real estate development, architectural design, and operational management to ensure efficient resource utilization and process optimization and ultimately achieve triple-bottom-line benefits in economic, social, and environmental dimensions. Labor, capital, and land are considered input variables because they represent the key factors of production in real estate. Labor represents human resource input, capital represents financial and technological equipment input, and land represents natural resource input. These factors collectively determine the quantity and quality of the output in real estate production. We selected the expected outputs from the economic, physical, and social dimensions based on how they reflect the economic, environmental, and social impacts of the real estate industry. Economic output includes market value, rental income, and other economic indicators. Physical output encompasses the quality, functionality, and lifespan of real estate structures, whereas social output focuses on the impact of real estate on communities, residents' lives, and public interests, such as accessibility, living quality, and social cohesion. Carbon dioxide emissions are considered an unexpected output because they represent environmental externalities associated with real estate production processes. Real-estate activity often involves energy consumption and waste emissions, which can negatively affect the atmosphere through climate change and air pollution. Therefore, considering carbon dioxide emissions as an unexpected output aims to reflect the adverse environmental impacts of real estate production and reduce these adverse effects by enhancing green efficiency to achieve environmental sustainability goals.

The input factors included labor force, capital, and land. The proxy variables included the number of employees and investments completed by real estate development enterprises and the total area of new housing construction. Desirable outputs comprised economic, physical, and social aspects. Economic output is represented by the sales of commodity houses, physical output by the completed housing area, and social output by taxes and additional contributions from real estate development enterprises. Carbon dioxide emissions are an undesirable output.

### Super-SBM model based on undesirable outputs

Considering the negative impact of the real estate industry on the ecological environment, this study used the super-SBM model with undesirable outputs to assess GPE in the real estate industry. First, the SBM model was used to determine whether the decision-making units were effective. Assuming that the kth ($k = 1, \ldots ,n$) decision-making unit contains m inputs $x_{k} = (x_{1k} , \ldots ,x_{mk} )$, *r*_1_ desirable outputs $y_{k}^{e} = \left( {y_{1k}^{e} , \ldots ,y_{{r_{1} k}}^{e} } \right)$, and *r*_2_ undesirable outputs $y_{k}^{u} = \left( {y_{1k}^{u} , \ldots ,y_{{r_{2} k}}^{u} } \right)$, all inputs and outputs are more significant than 0. Assuming constant returns to scale, the set of production possibilities is:

$$P_{1} (x,y^{e} ,y^{u} ) = \left\{ {\begin{array}{*{20}l} {x_{ik} = \sum\nolimits_{j = 1}^{n} {\lambda_{j} x_{ij} } + w_{i}^{ - } } \hfill & {i = 1, \ldots ,m} \hfill \\ {y_{sk}^{e} = \sum\nolimits_{j = 1}^{n} {\lambda_{j} y_{sj}^{e} } - w_{s}^{e} } \hfill & {s = 1, \ldots ,r_{1} } \hfill \\ {y_{qk}^{u} = \sum\nolimits_{j = 1}^{n} {\lambda_{j} y_{qj}^{u} } + w_{q}^{u} } \hfill & {q = 1, \ldots ,r_{2} } \hfill \\ \end{array} } \right\}$$

where λ denotes the weight vector, *w*_1_ is the input slack variable, *w*_2_ is the desirable output slack variable, and *w*_3_ is the undesirable output slack variable. The GPE calculation result (*GPE*_1_) in the real estate industry is as follows:

$$\min GPE_{1} = \frac{{1 - \frac{1}{m}\sum\nolimits_{i = 1}^{m} {\frac{{w_{i}^{ - } }}{{x_{ik} }}} }}{{1 + \frac{1}{{r_{1} + r_{2} }}\left( {\sum\nolimits_{s = 1}^{{r_{1} }} {\frac{{w_{s}^{e} }}{{y_{sk}^{e} }}} + \sum\nolimits_{q = 1}^{{r_{2} }} {\frac{{w_{q}^{u} }}{{y_{qk}^{u} }}} } \right)}}$$

The effective decision-making units are further distinguished. When *GPE*_1_ = 1, the decision-making unit is in an effective state, and the set of production possibilities for effective decision-making units must be redefined as follows:

$$P_{2} (x,y^{e} ,y^{u} ) = \left\{ {\begin{array}{*{20}l} {\tilde{x}_{i} \ge \sum\nolimits_{j = 1,j \ne k}^{n} {\lambda_{j} x_{ij} } \& \tilde{x}_{i} \ge x_{k} } \hfill & {i = 1, \ldots ,m} \hfill \\ {\tilde{y}_{s}^{e} \le \sum\nolimits_{j = 1,j \ne k}^{n} {\lambda_{j} y_{sj}^{e} } \& \tilde{y}_{s}^{e} \le y_{sk}^{e} } \hfill & {s = 1, \ldots ,r_{1} } \hfill \\ {\tilde{y}_{q}^{u} \ge \sum\nolimits_{j = 1,j \ne k}^{n} {\lambda_{j} y_{qj}^{u} } \& \tilde{y}_{q}^{u} \ge y_{qk}^{u} } \hfill & {q = 1, \ldots ,r_{2} } \hfill \\ \end{array} } \right\}$$

The GPE (*GPE*_2_) of the real estate industry in the effective decision unit is calculated as:

$$\min GPE_{2} = \frac{{\frac{1}{m}\sum\nolimits_{i = 1}^{m} {\frac{{\tilde{x}_{i} }}{{x_{ik} }}} }}{{\frac{1}{{r_{1} + r_{2} }}\left( {\sum\nolimits_{s = 1}^{{r_{1} }} {\frac{{\tilde{y}_{s}^{e} }}{{y_{sk}^{e} }}} + \sum\nolimits_{q = 1}^{{r_{2} }} {\frac{{\tilde{y}_{q}^{u} }}{{y_{qk}^{u} }}} } \right)}}$$

Moreover, the real estate industry's final GPE (*GPE*) is a product of *GPE*_1_ and *GPE*_2_:

### SDE

The SDE was used to reveal trends in the geographical center of gravity of GPE in the real estate industry. This method enables efficiency comparisons between various periods or regions, thereby helping to identify enhancement or deterioration trends. This assists in evaluating clustering and dispersion patterns in spatial distribution. This aids in shaping spatial strategies by providing insights into the development of efficiency centroids, thus guiding decision-makers in planning and policy formulation.

It was calculated as follows: First, the Miller cylindrical was used to transform the longitude and latitude coordinate system into a plane rectangular coordinate system. If $(x_{i} ,y_{i} )$ is used to represent the geographic coordinates of region *i* ($i = 1,2, \ldots ,n$) and $GPE_{i}$ is used to represent the GPE of the real estate industry in region *i*, then the geographical center of gravity $(x_{GPE} ,y_{GPE} )$ of the GPE in China’s real estate industry is:

$$x_{GPE} = \frac{{\sum\nolimits_{i = 1}^{n} {GPE_{i} \times x_{i} } }}{{\sum\nolimits_{i = 1}^{n} {GPE_{i} } }},\quad y_{GPE} = \frac{{\sum\nolimits_{i = 1}^{n} {GPE_{i} \times y_{i} } }}{{\sum\nolimits_{i = 1}^{n} {GPE_{i} } }}$$

The straight-line distance d between two adjacent geographical centers of gravity, $\left( {x_{GPE}^{t} ,y_{GPE}^{t} } \right)$ and $\left( {x_{GPE}^{t + 1} ,y_{GPE}^{t + 1} } \right)$ is:

$$d = \sqrt {\left( {x_{GPE}^{t + 1} - x_{GPE}^{t} } \right)^{2} + \left( {y_{GPE}^{t + 1} - y_{GPE}^{t} } \right)^{2} }$$

Next, we calculated the semi-major axis length (x-axis standard deviation) and the short half-axis length (y-axis standard deviation) of the standard deviation ellipse. Using $(\tilde{x}_{i} ,\tilde{y}_{i} )$ ($\tilde{x}_{i} = x_{i} - x_{GPE}$, $\tilde{y}_{i} = y_{i} - y_{GPE}$) to represent the relative coordinates of region i and the location of the center of gravity, let:

$$A = \sum\nolimits_{i = 1}^{n} {(GPE_{i} \times \tilde{x}_{i} )^{2} } - \sum\nolimits_{i = 1}^{n} {(GPE_{i} \times \tilde{y}_{i} )^{2} } ,\quad B = \sum\nolimits_{i = 1}^{n} {GPE_{i}^{2} \times \tilde{x}_{i} \times \tilde{y}_{i} }$$

Then, the elliptical azimuth $\theta$ is:

$$\theta = \arctan \frac{{A + \sqrt {A^{2} + 4B^{2} } }}{2B}$$

Finally, the standard deviations of the x and y axes were obtained as follows:

$$\begin{aligned} \sigma_{x} & = \sqrt {\frac{{\sum\nolimits_{i = 1}^{n} {(GPE_{i} \times \tilde{x}_{i} \times \cos \theta - GPE_{i} \times \tilde{y}_{i} \times \sin \theta )^{2} } }}{{\sum\nolimits_{i = 1}^{n} {GPE_{i}^{2} } }}} \\ \sigma_{y} & = \sqrt {\frac{{\sum\nolimits_{i = 1}^{n} {(GPE_{i} \times \tilde{x}_{i} \times \sin \theta - GPE_{i} \times \tilde{y}_{i} \times \cos \theta )^{2} } }}{{\sum\nolimits_{i = 1}^{n} {GPE_{i}^{2} } }}} \\ \end{aligned}$$

### Moran’s index

Using Moran’s index to analyze the spatial clustering trends of GPE in the real estate industry offers several benefits. This helps to identify whether there is spatial clustering or dispersion of GPE, which is crucial for understanding regional patterns. It guides spatial planning and policymaking by informing governments and relevant agencies about areas with low GPE, thereby prompting targeted interventions. It supports resource allocation and market positioning for businesses by directing investments toward regions with lower GPE, thereby enhancing market share and meeting local policy requirements.

First, the overall spatial agglomeration effect was tested. The overall Moran’s index was calculated as follows:

$$I = \frac{{\sum\nolimits_{i = 1}^{n} {\sum\nolimits_{j = 1}^{n} {W_{ij} (GPE_{i} - \overline{GPE} )(GPE_{j} - \overline{GPE} )} } }}{{S^{2} \sum\nolimits_{i = 1}^{n} {\sum\nolimits_{j = 1}^{n} {W_{ij} } } }}$$

where $S^{2} = \frac{1}{n}\sum\nolimits_{i = 1}^{n} {(GPE_{i} - \overline{GPE} )^{2} }$, $\overline{GPE} = \frac{1}{n}\sum\nolimits_{i = 1}^{n} {GPE_{i} }$, and $W_{ij}$ are the spatial weights used to measure the spatial association characteristics of the study objects. An inverse-distance weight matrix was used. Next, the agglomeration effect of the local regions and local Moran’s index were calculated as:

$$I_{i} = \frac{{GPE_{i} }}{{S^{2} }}\sum\nolimits_{j = 1}^{n} {W_{ij} GPE_{j} }$$

Based on the local Moran’s index, LISA were produced to determine agglomeration types in local areas.

### Theil index

Analyzing the GPE spatial disparity patterns in the real estate industry using the Theil index is meaningful because it quantifies regional inequalities. The index intuitively reflects the degree of inequality, showing the distribution of GPE across different areas and identifying high- and low-efficiency zone patterns. This analysis informs policymakers by providing insights into regional disparities, enabling governments to formulate targeted measures to promote development in less efficient areas, thus reducing regional disparities.

The primary sources were explored by comparing the contribution rate of the Theil index between the groups at different spatial partition scales. The Theil index, which was used to measure the overall disparity (*T*), was calculated as follows:

$$T = \frac{1}{n}\sum\nolimits_{i = 1}^{n} {\frac{{GPE_{i} }}{{\overline{GPE} }} \times \log \frac{{GPE_{i} }}{{\overline{GPE} }}}$$

where n is the number of provinces. Assuming that the entire region was divided into *K* sub-regions and the *k*th ($k = 1, \ldots ,K$) sub-region included *n*_*k*_ provinces, the inter-sub-regional disparity (*T*_*b*_) and intra-sub-regional disparity (*T*_*w*_) were:

$$\begin{aligned} T_{b} & = \sum\nolimits_{k = 1}^{K} {GPE_{k} \times \log \frac{{GPE_{k} }}{{{{n_{k} } \mathord{\left/ {\vphantom {{n_{k} } n}} \right. \kern-0pt} n}}}} \\ T_{w} & = \sum\nolimits_{k = 1}^{K} {GPE_{k} \times \left( {\sum\nolimits_{{i \in n_{k} }} {\frac{{GPE_{i} }}{{GPE_{k} }} \times \log \frac{{{{GPE_{i} } \mathord{\left/ {\vphantom {{GPE_{i} } {GPE_{k} }}} \right. \kern-0pt} {GPE_{k} }}}}{{{1 \mathord{\left/ {\vphantom {1 {n_{k} }}} \right. \kern-0pt} {n_{k} }}}}} } \right)} \\ \end{aligned}$$

where *GPE*_*k*_ was the ratio of the sum of the *k*th sub-regional GPE to the sum of the GPE of all regions, the inter-group contribution rate (*F*_*b*_) was the proportion of inter-sub-regional disparity to the overall disparity (*F*_*b*_ = *T*_*b*_*/T*), and the intra-group contribution rate (*F*_*w*_) was the proportion of intra-sub-regional disparity to the overall disparity (*F*_*w*_ = *T*_*w*_*/T*).

### RKDE and SMC

RKDE and SMC were used to analyze the dynamic evolution of GPE distribution in the real estate industry. RKDE, a nonparametric statistical method, is suitable for estimating the probability density function of data, particularly when dealing with small sample sizes or unknown data distributions, thus capturing potential distribution features without assuming specific distributions. SMC is adept at modeling data spatial dynamics, considering spatial correlations and dependencies. When studying GPE in the real estate industry, geographical location often plays a pivotal role, affecting efficiency through varying economic developmental levels, policy environments, and resource utilization patterns across regions. By employing SMC, it is possible to comprehend GPE evolutionary patterns across different areas and elucidate the influence of spatial correlations on this process. Integrating the RKDE and SMC enables a comprehensive and in-depth analysis of GPE dynamic evolutionary distribution, providing theoretical support and practical guidance for policymakers and strategic planning.

This study combined unconditional, space static, and spatial dynamic kernel density estimations to describe GPE distribution patterns and evolutionary trends in China's real estate industry from three aspects: time, space, and both combined. If *GPE*_*t*_ and *GPE*_*t*+*T*_ represent the GPE of the real estate industry in the current year and *T* years later in the region, respectively, *W* × *GPE*_*t*_ denotes the GPE in the current year between neighboring regions. Then, the unconditional (*f*_*UKD*_), space static (*f*_*SSKD*_), and spatial dynamic kernel densities (*f*_*SDKD*_), were calculated as follows:

$$\begin{aligned} f_{UKD} & = \frac{{f(GPE_{t} ,GPE_{t + T} )}}{{f(GPE_{t} )}} \\ f_{SSKD} & = \frac{{f(W \times GPE_{t} ,GPE_{t} )}}{{f(W \times GPE_{t} )}} \\ f_{SDKD} & = \frac{{f(W \times GPE_{t} ,GPE_{t + T} )}}{{f(W \times GPE_{t} )}} \\ \end{aligned}$$

where $f(u)$ and $f(u,v)$ denote the non-parametric estimation results of the one- and two-dimensional probability density functions, respectively.

The critical difference between spatial and traditional Markov chains is whether the spatial lag term is considered^43^. This study used two methods to explore the shifting GPE trends in China's real estate industry. First, we calculated a traditional Markov chain. Dividing the GPE of the real estate industry into *L* types using $n_{i}^{t}$ to denote the number of provinces belonging to type $i$ in year $t$, and $n_{ij}^{t,t + T}$ to denote the number of provinces that belong to type $i$ in year $t$ but become type $j$ after year $T$. Then, the transition probability matrix of GPE in the real estate industry in a particular region from type $i$ in year $t$ to type $j$ in year $t + T$ is:

$$P_{ij}^{t,t + T} = \frac{{\sum\nolimits_{2001}^{2020 - T} {n_{ij}^{t,t + T} } }}{{\sum\nolimits_{2001}^{2020 - T} {n_{i}^{t} } }},\quad i,j = 1,2, \ldots ,L$$

By adding the spatial lag term to the above equation, the $L \times L \times L$ order spatial Markov matrix was finally obtained.

## Results

### Spatial distribution pattern of GPE in China's real estate industry

#### Basic facts

**Figure 2:**
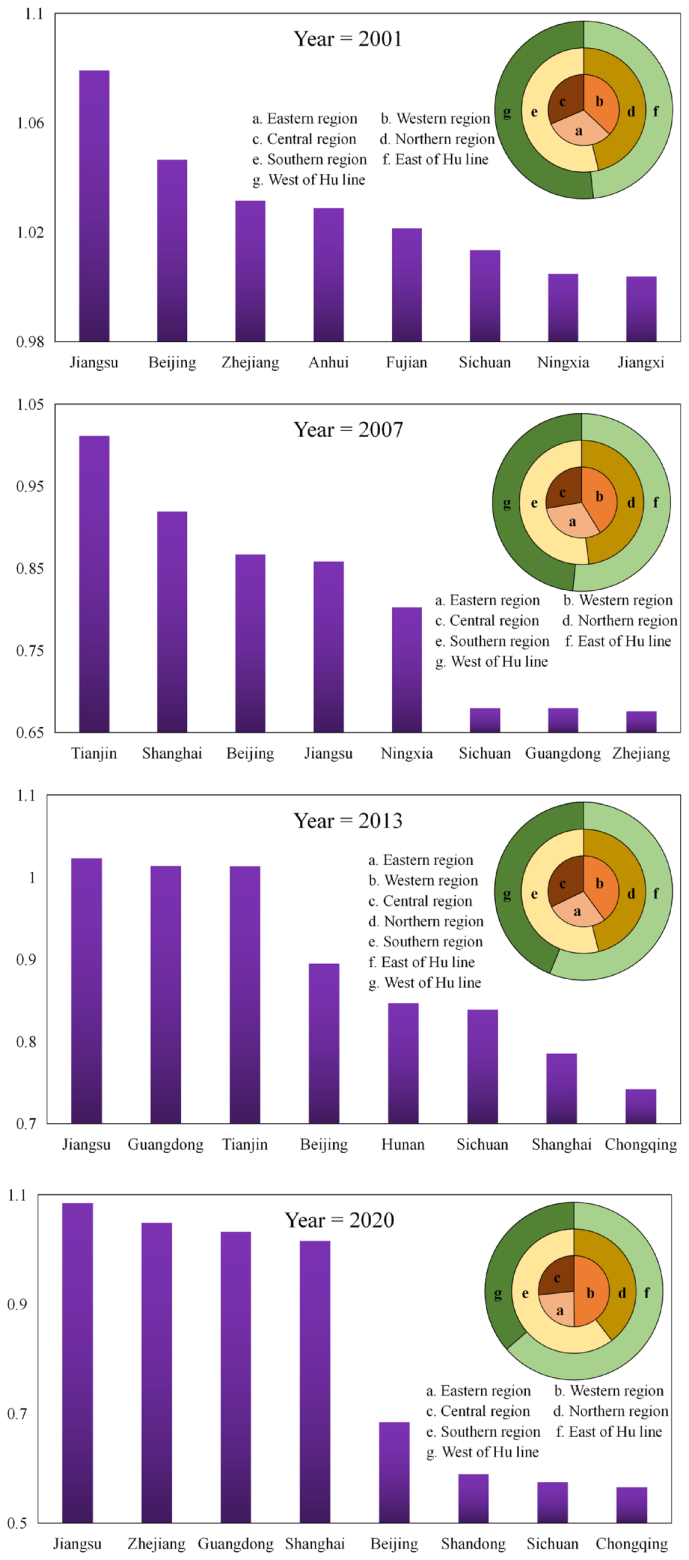
Descriptive statistics of GPE in China’s real estate industry in 2001, 2007, 2013, and 2020.

Figure 2 shows the top eight provincial GPE regional distribution statistics in China's real estate industry during the study period, which reveal the following spatial distribution characteristics.

The development of GPE fluctuated among different provinces over time. Throughout the study period, Jiangsu, Zhejiang, Shanghai, Beijing, Tianjin, and Guangdong were consistently among the top three provinces, with Jiangsu consistently leading GPE. This indicates solid regional dynamics among provinces with high efficiency levels. Considering effectiveness, only one province reached an influential state in 2007, increasing to three provinces in 2013, and four provinces in 2020. The efficiency disparity among the top four provinces continued to narrow, reflecting an ongoing increase in provinces with high efficiency and a gradual expansion trend.

Significant local disparities are evident. A trend of higher efficiency in the east and lower efficiency in the west was observed, with average efficiency values for the eastern, central, and western regions being 0.825, 0.701, and 0.704, respectively, in 2001. Although the eastern region led GPE within the real estate industry, overall efficiency across the three regions remained relatively balanced. However, after 2007, the efficiency disparity between the eastern, central, and western regions gradually widened. By 2020, the efficiency value of the eastern region reached 0.706, whereas the average efficiency value of both central and western regions was 0.356. The southern region consistently demonstrated higher efficiency from north to south than that of the northern region. A slight difference between the two occurred in 2006, with the efficiency of the southern region being only 6.98% higher than that of the northern region. Conversely, the most significant difference emerged in 2019, when the efficiency value of the southern region was 1.5 times that of the northern region. Regarding the Hu Line, the eastern provinces displayed higher efficiency. In 2001, the average efficiency value of provinces west of the Hu Line was slightly higher than that of provinces east of the Hu Line. However, the efficiency levels of provinces east of the Hu Line have consistently increased since then. By 2020, the efficiency level east of the Hu Line significantly surpassed that of the western provinces.

#### Evolution of the geographical center of gravity

**Figure 3:**
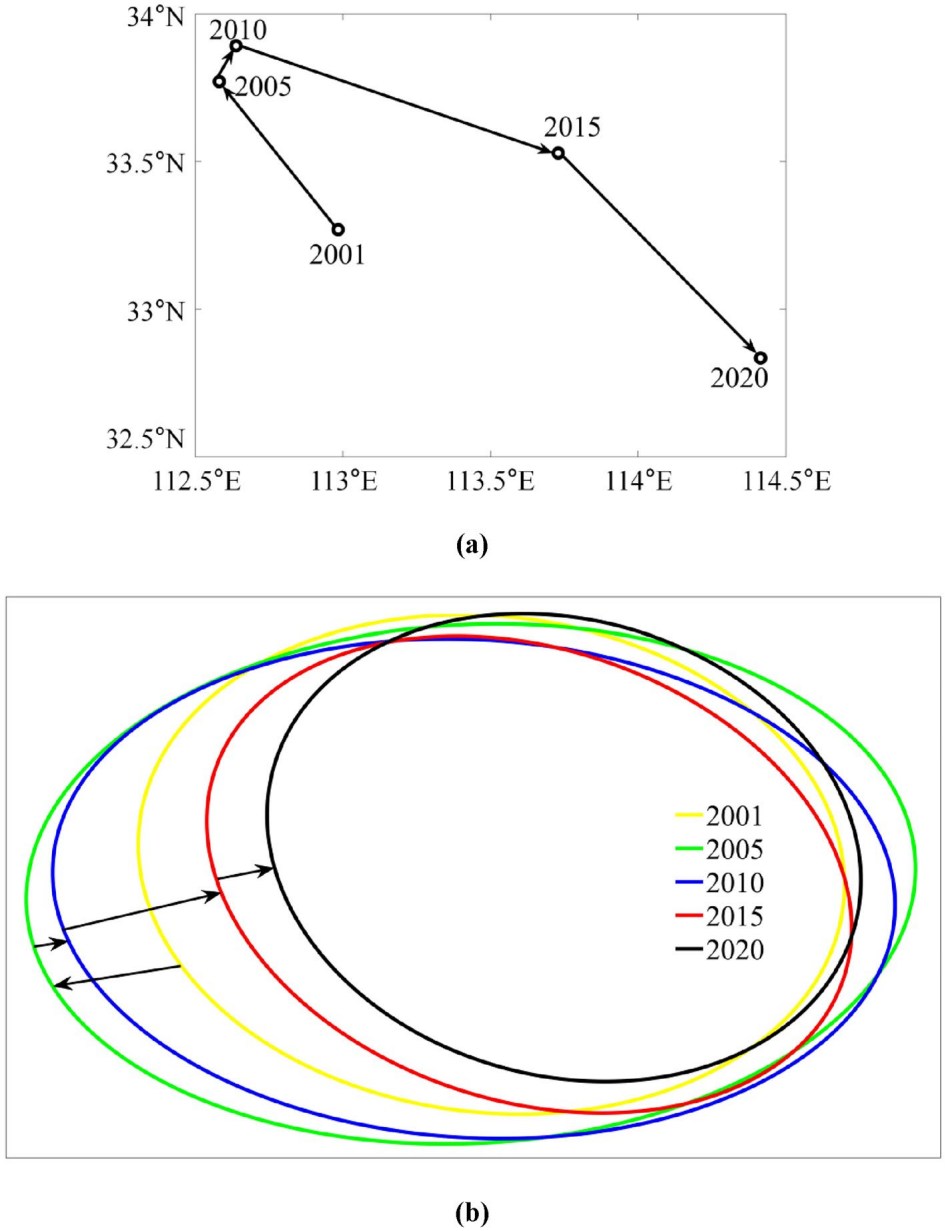
Spatial distribution of GPE in China’s real estate industry in 2001, 2005, 2010, 2015 and 2020: (**a**) Moving trajectories of the GPE gravity center in China’s real estate industry; and (**b**) SDE of GPE spatial distribution in China's real estate industry.

Figure 3 describes the GPE center of gravity trend in China's real estate industry based on the SDE.

Regarding location and directional movement, the center of gravity consistently resided in the Henan Province. Between 2001 and 2005, the geographical center shifted from Nanyang to Pingdingshan, Henan Province, moving northwestward over 71.45 km. From 2006 to 2010, it moved northeastward by 14.88 km while remaining centered in Pingdingshan, Henan Province. From 2011 to 2020, the center gradually shifted southeastward, spanning 127.80 km and 108.37 km, respectively. Consequently, the center was situated successively in Luohe and Zhumadian, Henan Province. These observations indicate a southeastward movement of the geographical center of GPE in China's real estate industry. While it moved northwestward from 2001 to 2010, the distance covered by the southeastward movement after 2010 significantly exceeded that of the previous northwestward movement.

In terms of the evolution of the SDE shape between 2001 and 2010, the long axis of the ellipse was oriented east–west, indicating a significant east–west disparity and minimal north–south difference in GPE within China's real estate industry during this period. The semi-major axis length measured 777.04 km in 2001, 976.10 km in 2005, and 924.99 km in 2010, suggesting a widening difference between the east and west. From 2011 to 2020, the ellipse's long axis gradually rotated toward the north–south, highlighting the north–south disparity in GPE in China's real estate industry. Eventually, it began to demonstrate a northwestward–southeastward difference by 2020. Notably, the length difference between the long and short axes of the ellipse gradually decreased during this period, indicating a weakening spatial orientation of GPE in China's real estate industry. Overall, China's real estate industry exhibited a point-symmetrical pattern, with Henan Province at its center, characterized by higher levels in the east and lower levels in the west. The east–west disparity prevailed as the primary characteristic before 2010, whereas the north–south disparity became prominent after 2010.

#### Spatial agglomeration evolution

**Figure 4:**
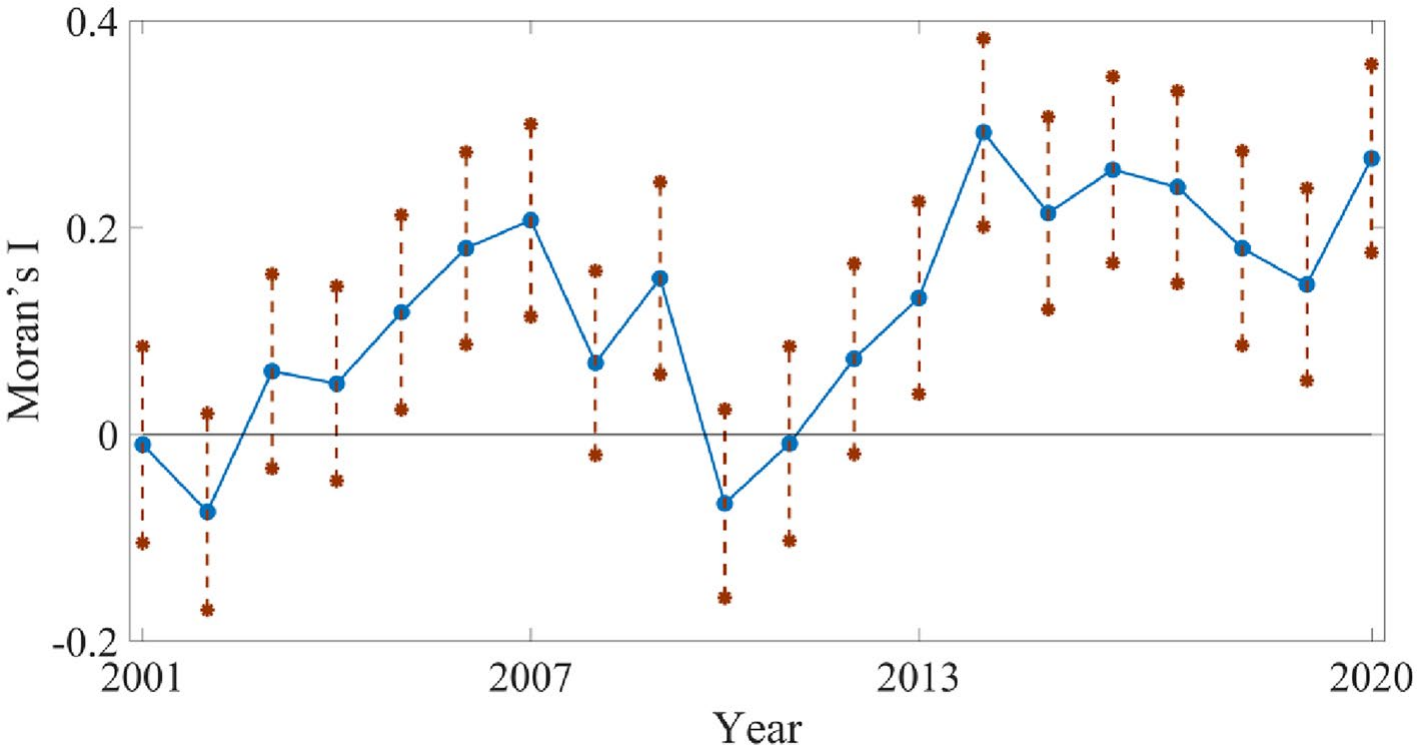
Moran’s index of GPE in China's real estate industry from 2001 to 2020.

Figure 4 shows the spatial agglomeration characteristics of GPE in China's real estate industry using the Moran’s index. The dashed line in the figure represents the 95% confidence interval. Values above the horizontal axis denote positive spatial agglomeration of GPE in the real estate industry, whereas those below indicate negative spatial agglomeration. A confidence interval encompassing the positive and negative values indicated a random spatial distribution of GPE. Except for 2001–2004, 2008, and 2010–2012, the Moran’s index passed the significance test at the 5% level, displaying positive values. This indicates that development of China’s real estate industry exhibited a positive spatial agglomeration. The Moran’s index ranged from 0.118 to 0.292, with a mean value of 0.198 (considering only values that passed the significance test), which suggest a robust spatial radiation effect within the real estate industry, indicating an average increase of 0.198% in neighboring areas when the efficiency level of the region increased by 1%. The spatial agglomeration of GPE in the real estate industry can be divided into two stages throughout the study period. There were alternating periods of insignificance and significance from 2001 to 2010, followed by a notably significant high-level agglomeration from 2011 to 2020.

**Figure 5:**
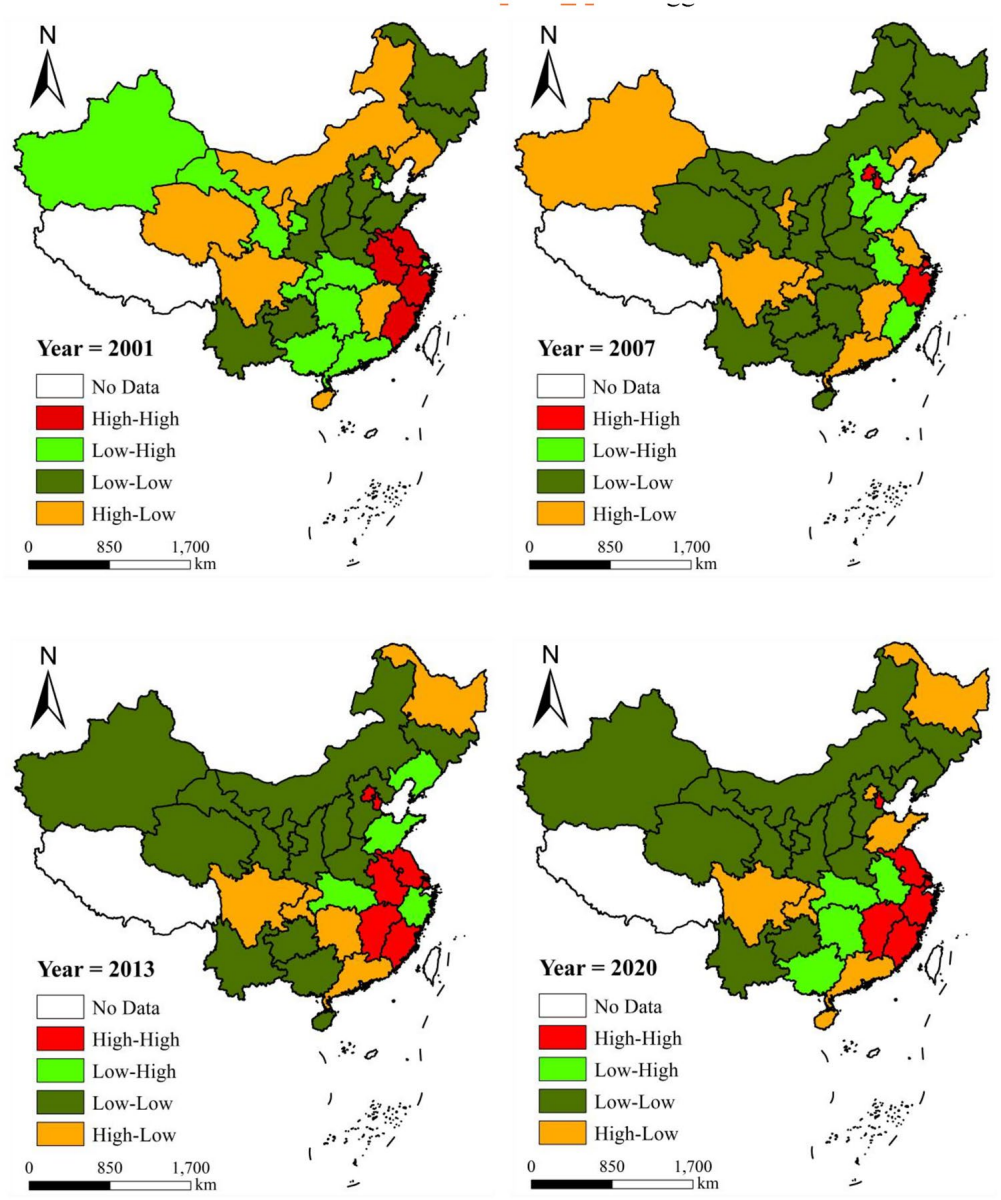
LISA agglomeration of GPE in China's real estate industry in 2001, 2007, 2013, and 2020.

Figure 5 presents the LISA results, detailing GPE local spatial agglomeration patterns in China. Positive spatial agglomeration refers to similar types of agglomeration characterized by high–high and low–low agglomerations. Conversely, negative spatial agglomeration refers to dissimilar types characterized by low–high and high–low agglomerations. First, positive spatial agglomeration remained the primary characteristic of local areas. In 2001, the number of provinces exhibiting negative agglomeration was slightly higher than the number of provinces exhibiting positive agglomeration. However, the number of provinces with positive agglomeration consistently increased thereafter. From 2007 to 2020, > 60% of the provinces nationwide displayed positive agglomeration, peaking in 2013 at 70%. Second, considering specific local areas, the eastern coastal and middle reaches of the Yangtze River region primarily showed high–high agglomeration. The middle Yellow River, northeastern, southwestern, and northwestern regions primarily exhibited low–low agglomeration. The number of provinces showing high–high and low–low agglomerations in the northern and southern coastal regions was evenly split. Specific provinces such as Beijing, Tianjin, and Fujian predominantly displayed high–high agglomeration, whereas Hebei and Hainan tended toward low–low agglomeration.

### GPE spatial disparity patterns in China's real estate industry

#### Overall spatial disparities and evolution

**Figure 6:**
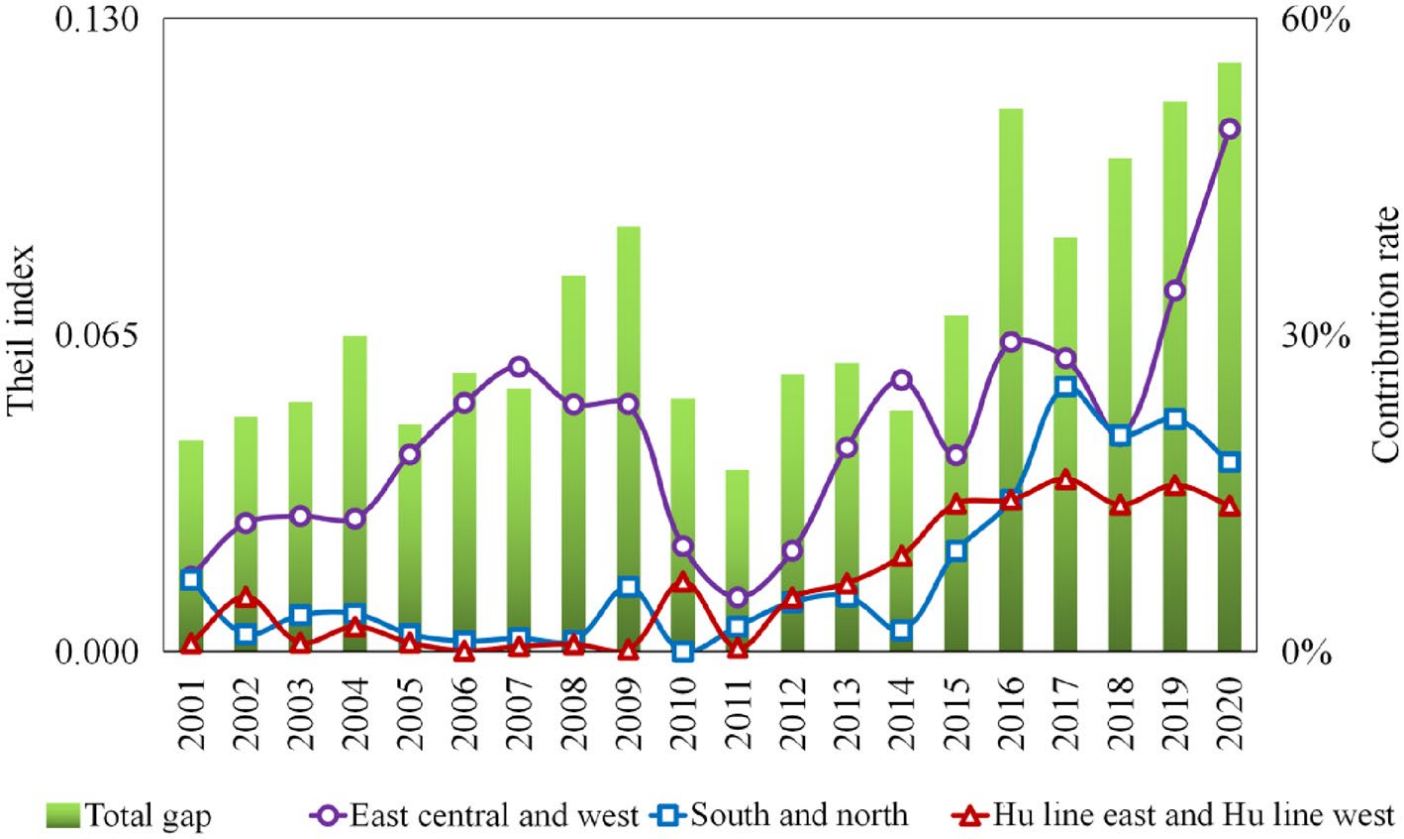
Decomposition of the Theil index of GPE of the real estate industry in different regions in China from 2001 to 2020.

Figure 6 shows the overall GPE spatial disparity in China's real estate industry from 2001 to 2020.

First, the overall disparity in GPE exhibited an "N"-shaped trend during the study period. The Theil index increased from 0.043 in 2001 to 0.087 in 2009, indicating an average annual growth rate of 9.1%. Subsequently, from 2009 to 2011, it decreased by 57.1%, reaching a GPE of 0.549 in 2011, which marked the lowest overall spatial disparity. This suggests that low-level convergence is a significant factor that hinders GPE improvement in the real estate industry. However, from 2011 to 2020, the Theil index increased notably from 0.085 in 2017 to 0.121 in 2020, contributing to the continued expansion of overall spatial disparity.

Second, intra-regional disparities emerged as the primary source of overall spatial disparity in GPE in China’s real estate industry. Throughout the study period, the maximum contribution rates of intergroup disparity for the east–west, north–south, and east–west directions of the Hu Line were 49.5, 25.1, and 16.4%, respectively. All three spatial division scales exhibited intragroup disparity contribution rates > 50%. This underscores the necessity of investigating the causes of spatial disparity patterns in GPE within China's real estate industry, starting from the perspective of intra-regional disparities.

Third, east–west and north–south disparities were crucial components of the GPE disparity pattern. Regarding the east–west direction, the contribution rate of the inter-group disparity displayed an "N"-shaped trend throughout the study period, with 2007 and 2011 as inflection points. It consistently held the top position, particularly from 2016 to 2020, exceeding 20% with a noticeable growth trend. Comparatively, when analyzing the north–south direction along the east–west axis of the Hu Line, the contribution rate of the disparity between these two groups alternated as the second-ranking factor. Notably, from 2017 to 2020, the north–south disparity emerged as a secondary characteristic of China's real estate industry.

#### Local regional disparities and evolution

**Figure 7:**
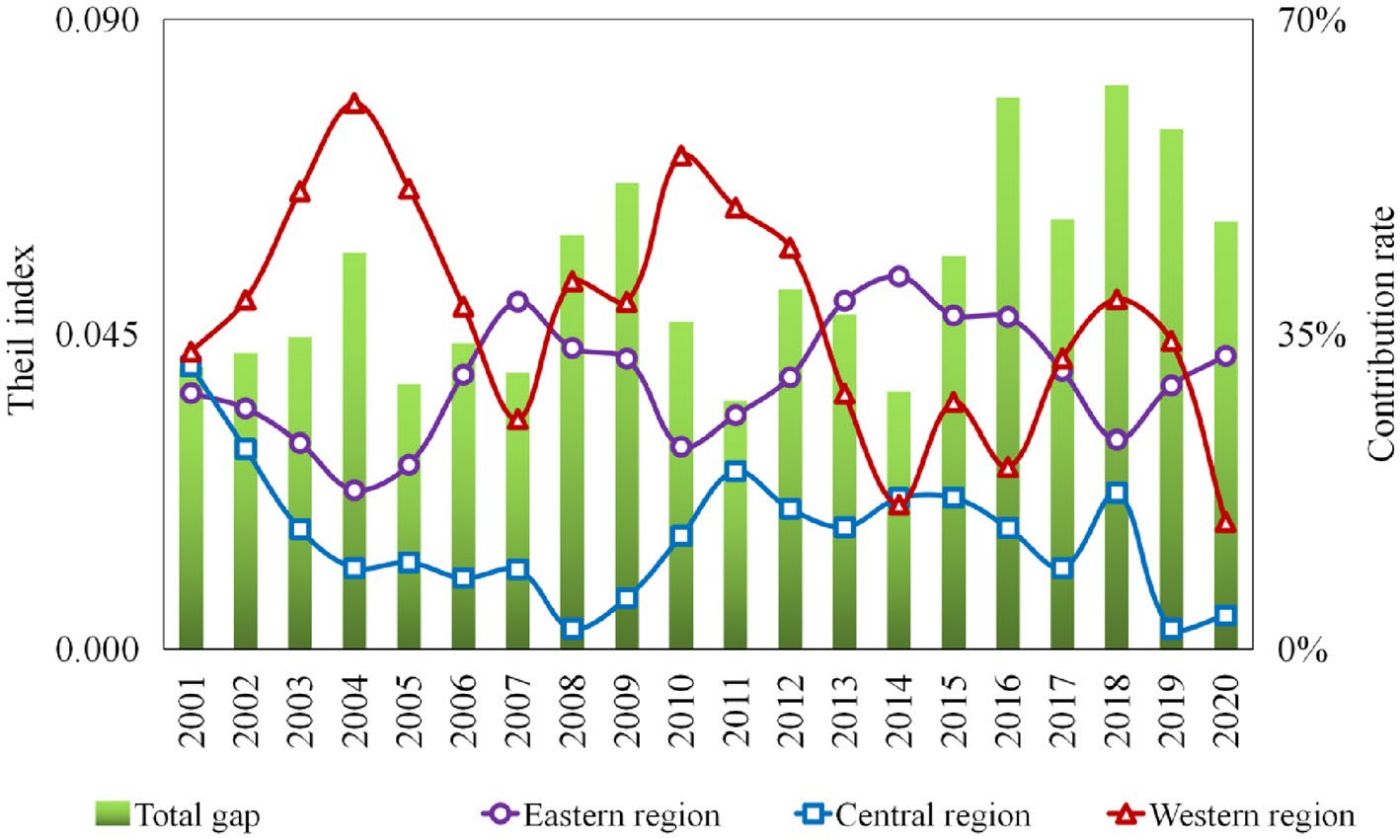
Decomposition of the Thiel index of real estate GPE in eastern, central, and western regions from 2001 to 2020.

Figure 7 illustrates the internal disparity in the three east–west regions based on the Theil index. From 2001 to 2020, the intergroup Theil index fluctuated between 0.035 and 0.081. Notably, the central region consistently exhibited the least disparity, with a disparity rate of 12.6% during the study period. In contrast, the disparity rate in the western region followed an "M"-shaped trend, with inflection points observed in 2004, 2007, and 2010. During this period, it consistently surpassed that of the eastern region, from 2001 to 2012. However, from 2013 to 2020, the disparity in the eastern region exceeded that in the western region.

**Figure 8:**
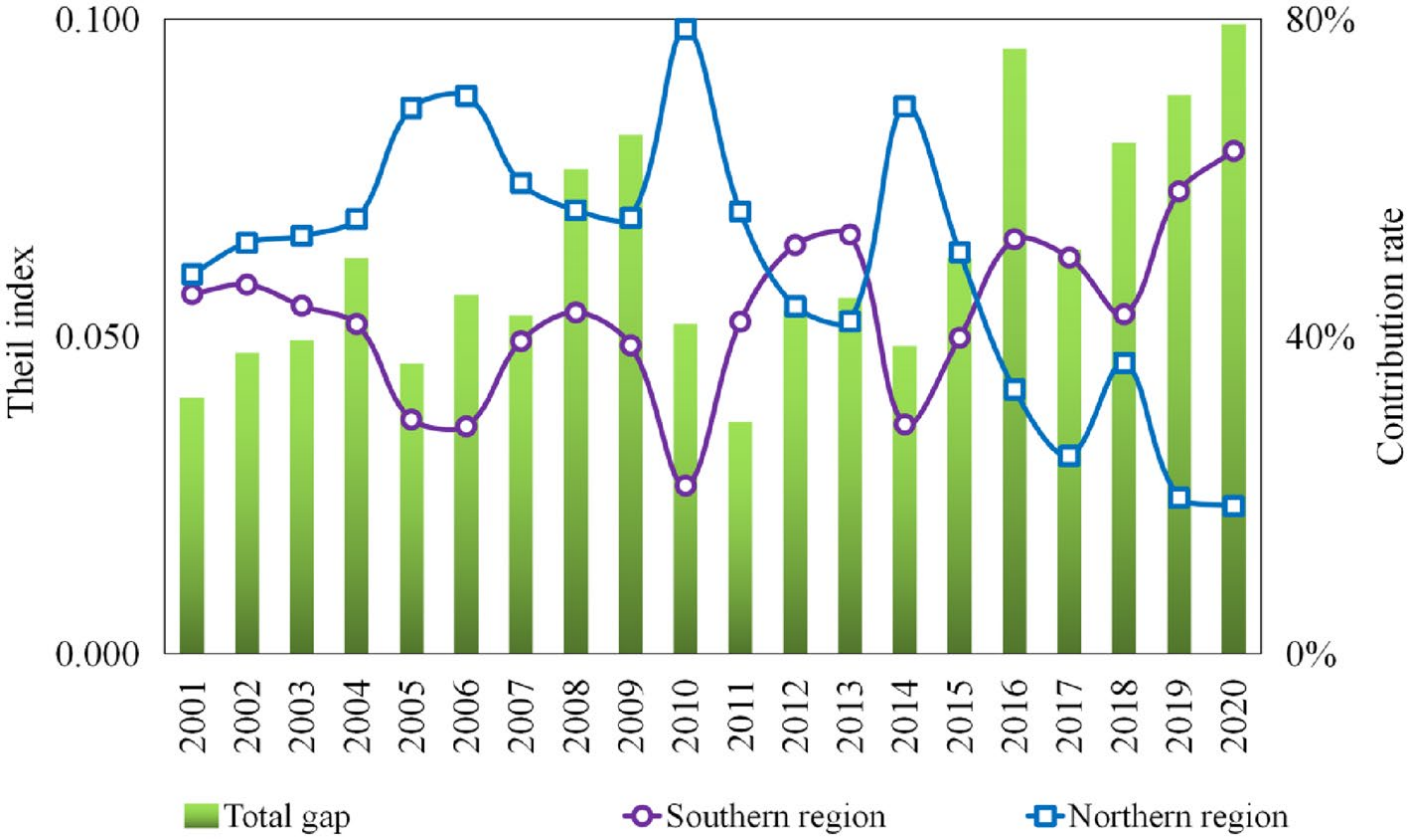
Decomposition of the Thiel index of real estate GPE in southern and northern regions from 2001 to 2020.

Figure 8 illustrates the internal disparity between the two primary north–south regions. The north–south intra-regional Theil index fluctuated between 0.037 and 0.099 throughout the study period. Interestingly, the disparity within this axis gradually emerged as the primary characteristic of China's real estate industry, superseding that in the east–west. Considering 2011 as a pivotal point, the disparity contribution rate of the northern region surpassed that of the southern region between 2001 and 2011. In 2010, the disparity within the northern region was most pronounced, with a disparity contribution rate of 78.8%. However, from 2012 to 2020, the disparity in the southern region exceeded that in the northern region. In particular, after 2014, the disparity contribution rate in the southern region increased markedly, reaching 63.4% in 2020.

**Figure 9:**
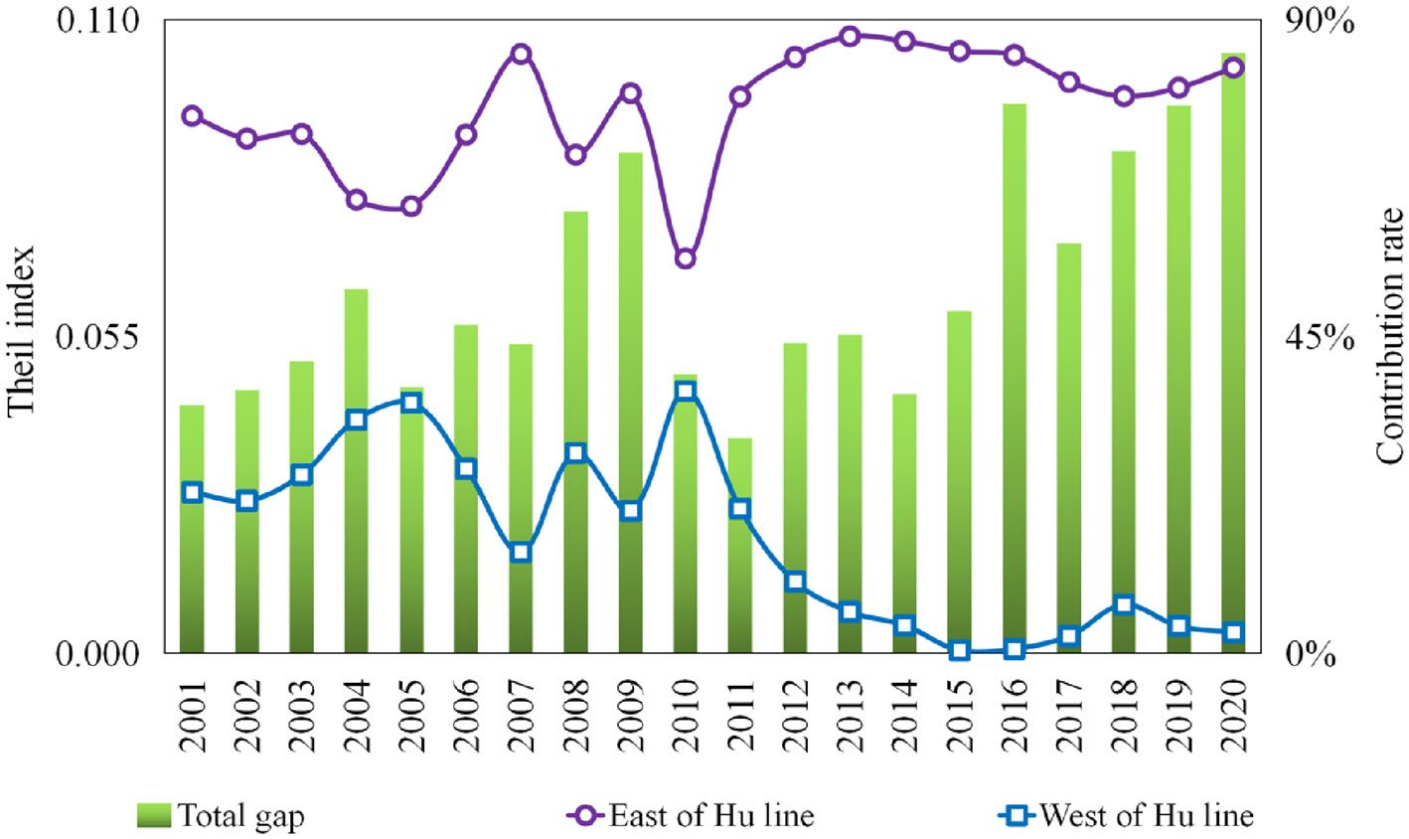
Decomposition of the Thiel index of real estate GPE east and west of the Hu line from 2001 to 2020.

Figure 9 shows the internal disparity between the two regions divided by the Hu Line (east and west). Across the study period, the Theil index within the east–west grouping of the Hu Line exhibited a phased growth trend, rising from 0.043 in 2001 to 0.087 in 2009, marking a 115.1% increase from 2010 to 2020, reaching a peak of 0.104 in 2020. Throughout this period, the contribution rate of the internal disparity east of the Hu Line fluctuated between 56.1 and 87.6%, significantly surpassing that west of the Hu Line. Contribution rates increased significantly after 2010.

### GPE dynamic distribution evolution in China's real estate sector

#### RKDE analysis

To investigate GPE distribution evolution in China's real estate industry over time and space, a three year period T was considered. RKDE analysis was employed to illustrate the unconditional, space static, and spatial dynamic kernel densities.

**Figure 10:**
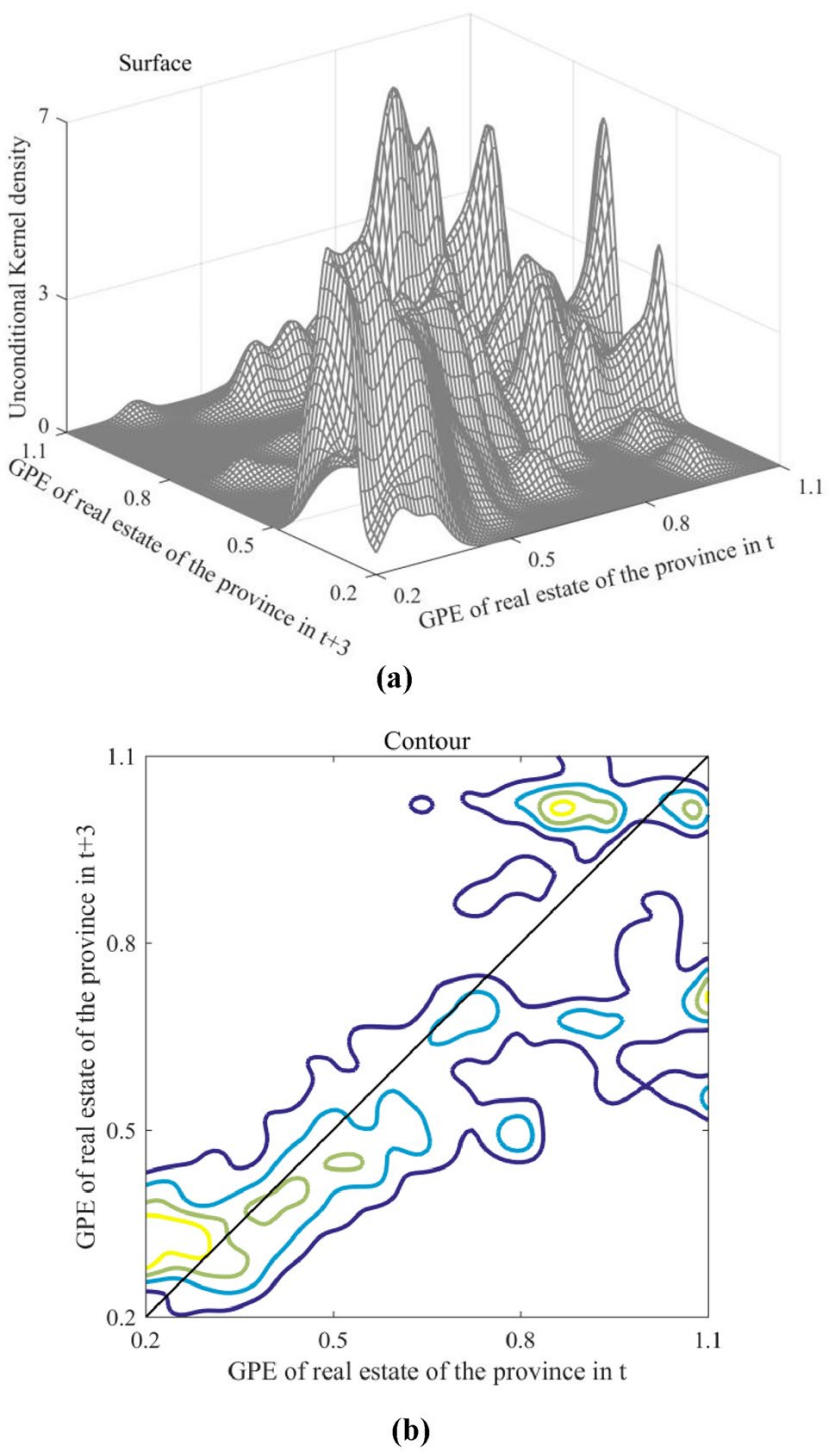
Unconditional kernel density results: (**a**) Surface and (**b**) Contour.

Unconditional kernel density illustrates GPE dynamic evolution in China's real estate industry across two periods. In Fig. 10, the horizontal axis represents GPE in the real estate industry for the first period (2001–2017), and the vertical axis represents the same for the second period (2004–2020). The depth axis depicts the results of the kernel density estimation, signifying the conditional probability density from year t to year t + 3.

Efficiency levels in China's real estate industry have exhibited heterogeneity over time. First, the low-GPE regions demonstrated time convergence, as observed in the symmetrically distributed probability density curves around the diagonal for efficiency values between 0.2 and 0.45. This suggests a minimal change in GPE in these provinces from year t to t + 3. For instance, Shanxi, Guizhou, and Hebei showed mean GPE values of 0.322, 0.365, and 0.399, respectively, fluctuating by no more than 3.8%. Second, the trend in middle-level regions may have been more optimistic. The probability density curve, predominantly positioned below the diagonal for efficiency values between 0.45 and 0.75, indicates a decline in GPE in these provinces from year t to t + 3. For example, Jilin, Qinghai, Inner Mongolia, and Xinjiang displayed GPE values of 0.472, 0.518, 0.573, and 0.636, respectively, in the first period, with declines of 8.6, 19.3, 19.4, and 14.8% in the second. Third, polarization was observed in high-GPE regions. With efficiency values ranging from 0.75 to 1.1, the probability density curves on either side of the diagonal were more distant, signifying more significant fluctuations in GPE in the high-GPE provinces from year t to t + 3. These provinces are divided into two categories: positive and negative. Provinces such as Shanghai and Guangdong experienced favorable growth rates of 6.0 and 7.3%, respectively, whereas Sichuan, Ningxia, and Beijing exhibited negative growth rates of 5.3, 6.6, and 6.7%, respectively.

**Figure 11:**
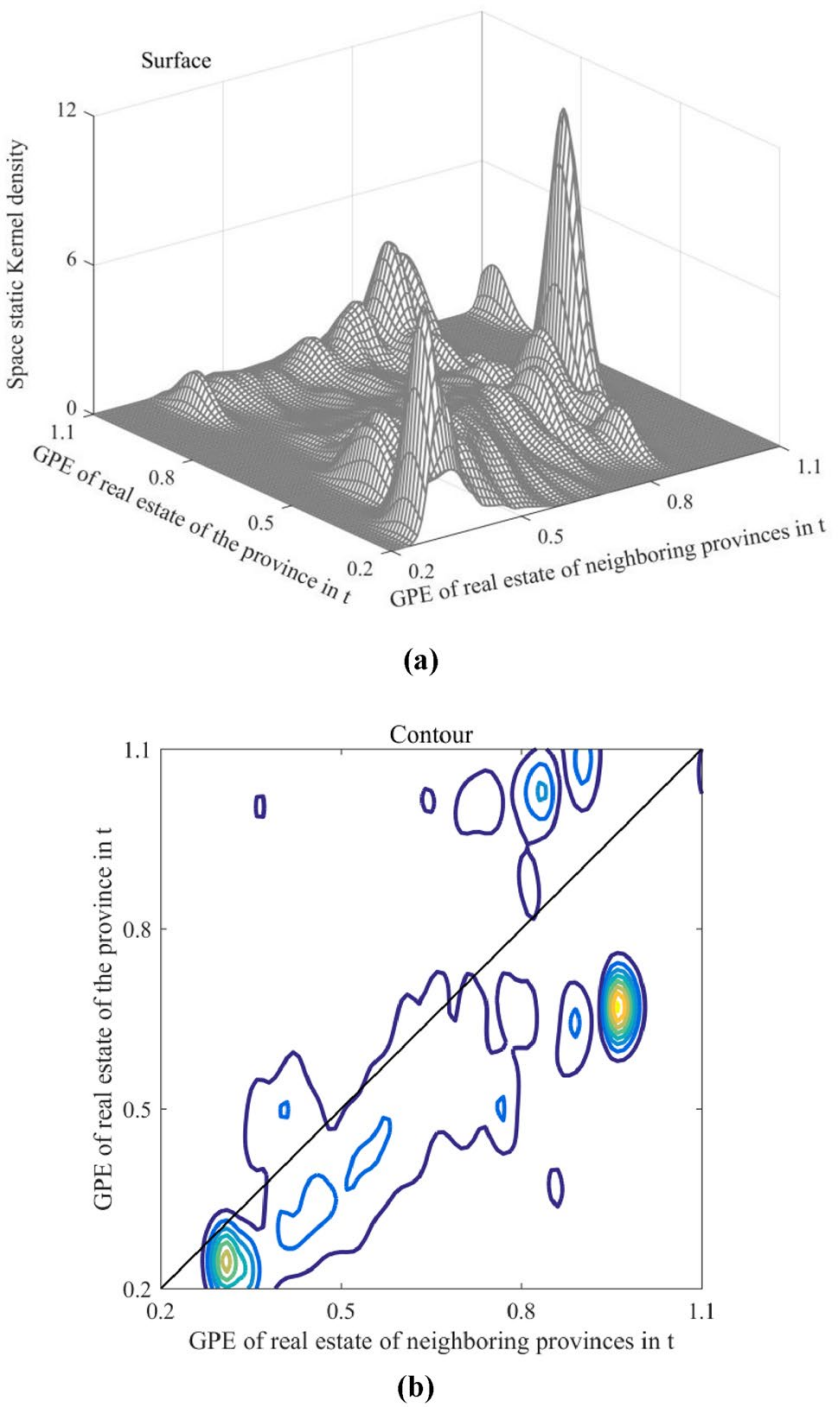
Space static kernel density results: (**a**) Surface; and (**b**) Contour.

The space static kernel density illustrates the dynamic evolution of GPE in China's real estate industry among neighboring regions. The horizontal axis in Fig. 11 represents the GPE of this region, whereas the longitudinal axis represents the GPE of the neighboring regions. The vertical axis displays the estimated results of the conditional probability density from the neighboring regions to this specific region.

There has been convergence in GPE in China's real estate industry. First, the club convergence of GPE in the real estate industry in the low-GPE regions is evident. When efficiency values range from 0.2 to 0.45, the contour lines on the graph form a circle centered around the efficiency value of 0.3. The probability density curve lies entirely below the diagonal, indicating that China's real estate industry exhibited low-level agglomeration and lower efficiency levels in neighboring regions hindered regional development. For instance, in the northwestern region, due to relatively low efficiency levels in Gansu and Qinghai during the study period, Ningxia's GPE declined from 0.676 in 2011 to 0.441 in 2020 due to the influence of neighboring regions. Second, the club convergence of GPE in the middle-level regions could be more robust. The probability density curve is sparse and primarily lies below the diagonal when the efficiency value ranges from 0.45 to 0.75, indicating that the development of medium-level provinces was susceptible to the constraints of neighboring areas, resulting in weak convergence. The mean value of GPE for economic zones in the middle reaches of the Yellow and Yangtze Rivers decreased from 0.736 to 0.363 during the study period, leading to a widening disparity in efficiency levels between the two economic zones. Third, GPE in the high-GPE region exhibited a multipolar trend. When efficiency values range from 0.75 to 1.1, there are at least two dense central circles on both sides of the diagonal in the diagram, indicating polarization in the high-GPE provinces. Neighboring areas had both promoting and inhibiting effects on the region. For example, Beijing, Shanghai, and Zhejiang exhibited facilitating effects, whereas Anhui, Fujian, and Jiangxi displayed suppressive effects.

**Figure 12:**
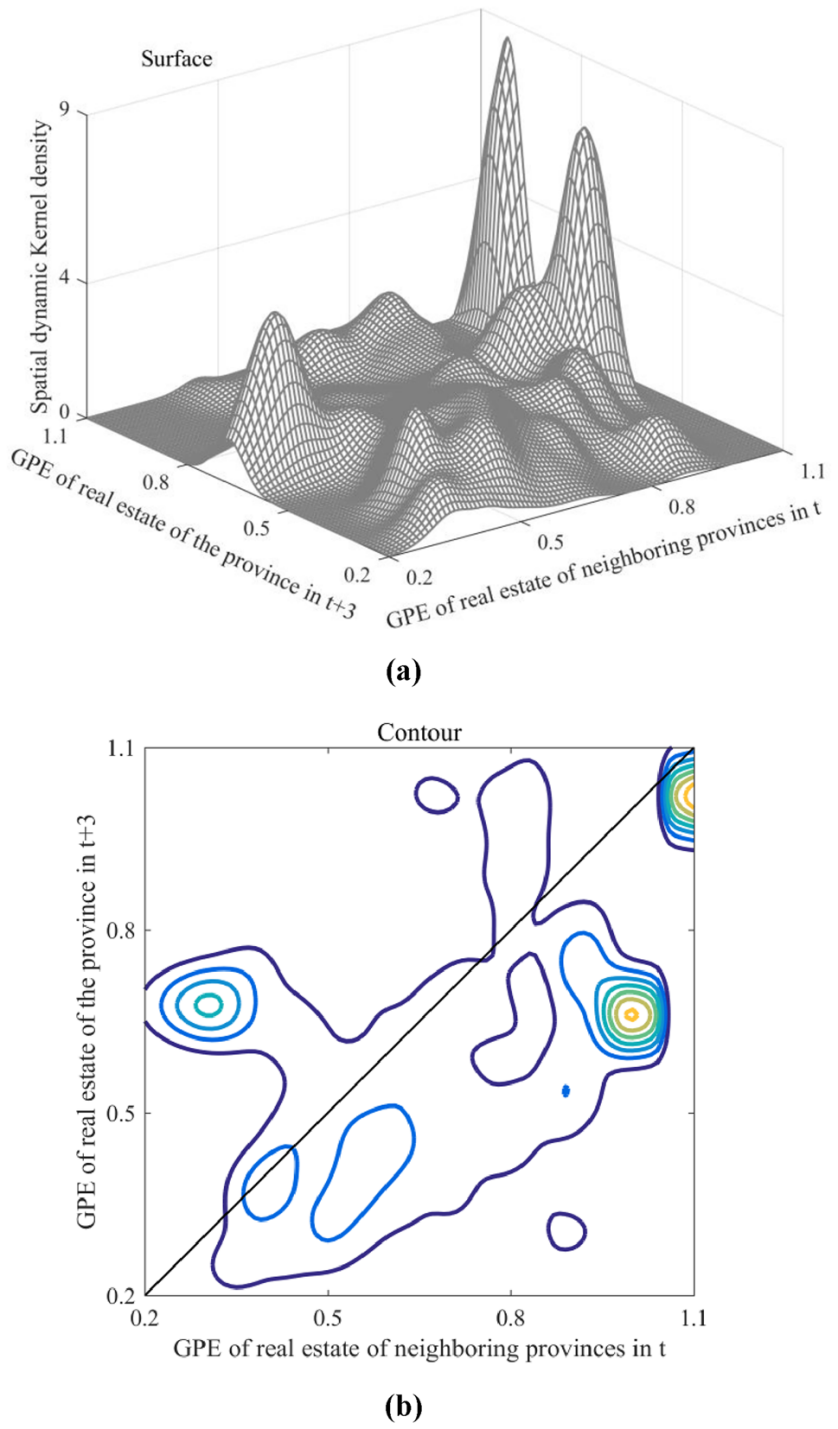
Spatial dynamic kernel density results: (**a**) Surface; and (**b**) Contour.

The spatial dynamic kernel density illustrates the evolution of GPE in neighboring regions in the two periods. The horizontal axis in Fig. 12 denotes the real estate industry's GPE in neighboring regions during the first period, whereas the longitudinal axis represents the regional GPE in the second period. The vertical axis indicates the conditional probability density from neighboring regions in the first period to regions in the second period.

The GPE spatial spillover effect exhibited multiple characteristics. First, this effect was positive in the low-GPE regions. When the efficiency levels of adjacent areas in the first period ranged from 0.2 to 0.4, the efficiency values of the region in the second period decreased primarily between 0.6 and 0.8. For example, the real estate industry in large cities affected northern coastal economic zones. Some production factors from Hebei and Shandong were transferred to Beijing and Tianjin, causing the agglomeration of elements that fostered growth in their real estate industries. Conversely, the loss of these factors resulted in a significant decline in efficiency in Hebei and Shandong. Second, the GPE of the real estate industry in the medium-level regions primarily exhibited spatial inhibition. When efficiency levels of neighboring regions ranged from 0.4 to 1.0, the contours primarily appeared below the diagonal, with only a few provinces showing the possibility of progressing upward. Provinces that were significantly inhibited were primarily located in the middle reaches of the Yellow and Yangtze River economic zones. In contrast, these boosts were primarily observed in the eastern coastal economic zones. Third, the spatial spillover effect of GPE in high-GPE regions showed continuity. When the efficiency level of neighboring regions ranged between 1.0 and 1.1, the central points of the contour lines aligned with the diagonal and exhibited a symmetrical distribution, indicating club convergence in the high-GPE region, which remained consistent over time. This phenomenon was more pronounced in the Yangtze River Delta city cluster.

#### SMC analysis

RKDA illustrates the GPE distribution dynamics in the real estate industry based on continuous variables. Simultaneously, SMC measures the transition probability in different states using discrete variables. These methods correspond to nonparametric and parametric measurement techniques, respectively, and their conclusions mutually verify and complement each other. Using the quartile principle, the real estate industry’s GPE across the 30 provinces during the study period was categorized into four types: low, medium, high, and very high. To compare the impact of temporal variations on transition probabilities, Table 1 presents a Markov state matrix with spans of 1, 3, and 5 years.

**Table 1:** The spatial Markov transfer matrix of GPE in the real estate industry under different periods from 2001 to 2020.

Type		T = 1				T = 3				T = 5			
		1	2	3	4	1	2	3	4	1	2	3	4
Non-space	1	0.783	0.178	0.026	0.013	0.699	0.228	0.052	0.022	0.642	0.225	0.100	0.033
	2	0.188	0.587	0.218	0.008	0.269	0.412	0.261	0.059	0.267	0.371	0.257	0.105
	3	0.052	0.177	0.569	0.203	0.066	0.248	0.453	0.234	0.107	0.265	0.397	0.231
	4	0.000	0.008	0.250	0.742	0.000	0.042	0.305	0.653	0.019	0.067	0.317	0.596
1	1	0.895	0.105	0.000	0.000	0.647	0.294	0.000	0.059	0.600	0.333	0.000	0.067
	2	0.105	0.579	0.290	0.026	0.265	0.529	0.206	0.000	0.267	0.367	0.333	0.033
	3	0.000	0.290	0.500	0.211	0.088	0.118	0.588	0.206	0.133	0.200	0.367	0.300
	4	0.000	0.026	0.211	0.763	0.000	0.088	0.177	0.735	0.000	0.100	0.300	0.600
2	1	0.737	0.237	0.000	0.026	0.677	0.235	0.000	0.088	0.600	0.300	0.033	0.067
	2	0.237	0.553	0.079	0.132	0.235	0.529	0.059	0.177	0.333	0.300	0.167	0.200
	3	0.053	0.263	0.316	0.368	0.118	0.294	0.471	0.118	0.067	0.533	0.267	0.133
	4	0.000	0.079	0.263	0.658	0.029	0.088	0.206	0.677	0.033	0.133	0.167	0.667
3	1	0.790	0.158	0.026	0.026	0.706	0.147	0.118	0.029	0.600	0.200	0.133	0.067
	2	0.158	0.500	0.316	0.026	0.177	0.529	0.206	0.088	0.167	0.467	0.233	0.133
	3	0.026	0.263	0.368	0.342	0.088	0.206	0.353	0.353	0.133	0.267	0.267	0.333
	4	0.026	0.079	0.290	0.605	0.029	0.118	0.324	0.529	0.100	0.067	0.367	0.467
4	1	0.842	0.158	0.000	0.000	0.794	0.177	0.000	0.029	0.700	0.233	0.000	0.067
	2	0.132	0.632	0.132	0.105	0.118	0.441	0.177	0.265	0.167	0.400	0.200	0.233
	3	0.000	0.263	0.368	0.368	0.177	0.118	0.235	0.471	0.133	0.133	0.133	0.600
	4	0.026	0.079	0.184	0.711	0.000	0.324	0.206	0.471	0.067	0.300	0.233	0.400

1. Low-GPE provinces; 2. Medium GPE provinces, 3. Relatively high-GPE provinces, 4. High-GPE provinces.

When the spatial spillover effect of GPE in the real estate industry is not considered, three distinct characteristics define development within regional real estate sectors.

The primary diagonal elements within the state transition probability matrix exhibited higher values than those in other positions, indicating club convergence in China's real estate industry GPE. For example, if a region falls within a specific GPE type in a given year, the likelihood of remaining in that category one year later ranges from 56.9 to 78.3%. This reflects strong GPE persistence. Notably, the probability values for both low- and high-GPE exceeded 70%, signifying a tendency in the Chinese real estate industry for emergence of a 'Matthew effect' characterized by polarization between high- and low-GPE levels.

Additionally, nonzero elements across the matrix primarily exist on both sides of the primary diagonal, indicating prevalent transitions among different GPE levels. Throughout the study period, medium-GPE regions tended to transition toward both low- and relatively high-GPE regions, with an increasing probability of moving toward relatively high-GPE regions and a declining trend in transitioning toward low-GPE regions as the span extended. Regarding transition patterns, relatively high-GPE regions were more likely to transition to medium- and high-GPE categories. However, the probability of transitioning to a high-GPE, albeit slightly more prominent, suggests an observable upward trend in GPE within China's real estate industry.

Third, the primary diagonal element notably decreased as the period was extended, whereas the elements in other positions gradually increased. This trend suggests that GPE mobility is enhanced across regions over time. With the span extending from one to five years, the probability of medium- and relatively high-GPE regions maintaining their status decreased from 58.7 and 56.9% to 37.1 and 39.7%, respectively. At the 5-year mark, more than half of the regions experiencing medium- and relatively high-GPE experienced horizontal transitions.

By including the spatial lag term in the traditional Markov state matrix, most elements in the transition probability matrix were altered. This alteration suggests a spatial spillover effect on GPE transitional trends; changes in regional GPE in the current period are influenced by development of surrounding areas in the previous period. Despite club convergence characteristics in GPE in the real estate industry, considering spatial factors, convergence varied across different types.

First, low-GPE regions remained consistently stable regardless of the type of spatial lag term applied. When comparing low-GPE neighbors to high-GPE neighbors, the region had an 89.5% probability of maintaining low-GPE with the former and 84.2% with the latter. This situation tends to result in susceptibility to low-level traps with low-GPE neighbors, whereas a higher likelihood of polarization arises with high-GPE neighbors. Additionally, an increasing spatial lag term type can amplify the probability of an upward shift in low-GPE regions.

Second, high-level GPE dominance is moderated by the inclusion of spatial factors, whereas a more extended duration disrupts high-GPE club convergence. The probability of high-GPE regions maintaining their status throughout the study period ranged from 40.0 to 76.3%, suggesting that development of high-GPE regions lacked complete dominance and was susceptible to influence from surrounding areas. In particular, the inhibitory effect originated from the medium- and relatively high-GPE regions.

Third, regardless of neighboring spatial lag type, the spatial mobility of medium- and relatively high-GPE regions was robust, indicating an apparent spatial spillover effect. Upon comparing the magnitudes of the elements in the transition probability matrix, it became evident that having low-GPE neighbors hampers efficiency improvement in the region, whereas having high-GPE neighbors encourages an upward transition of efficiency levels. Additionally, the passage of time accentuates the transition of GPE from one type to another.

## Discussion

The results of spatial distribution characteristics showed the following: First, environmental regulations guide green real estate. Under these regulations, the real estate industry has transitioned from an extensive to an intensive model, with the higher intensity of environmental regulations in the eastern region being the key reason for GPE disparity between the eastern and central regions of the real estate industry^39,40^. Second, salary level drives real estate consumption. Since its reform and opening-up, the southern regional economy has undergone rapid development. Guided by market resource allocation, wealth has accumulated significantly in the southern region. Improvements in salaries provide sufficient funds for consumers to purchase houses, contributing to capital accumulation for the development of the real estate industry in the southern region^45^. Third, the real estate industry depends on population density. Provinces with high population densities, substantial population capacities, and advanced infrastructure exhibited relatively well-developed GPE in the real estate industry. As a geographical dividing line for population density in China, the efficiency disparity between the eastern and western regions of the Hu Line provides robust evidence for this third conclusion^35,36^.

The spatial agglomeration evolutionary trend revealed several significant aspects. First, China's real estate industry aligns with green technology innovation in its spatial agglomeration characteristics. Environmental regulations drive the development of green real estate, whereas technological innovation supports improvements in GPE within the industry^46,47^. Second, from 2001 to 2010, the real estate industry primarily focused on vigorously developing ordinary and affordable housing aligned with urbanization efforts. However, its spatial radiation effects were only partially evident during this period^48^. Third, there was a developmental lag in the real estate sector, and accumulations from 2001 to 2010 paved the way for a boom in the industry from 2010 to 2015, with a rapid rise in the spatial radiation effect^49,50^. Finally, during the adjustment period of 2016–2020, the central government implemented various policies related to purchase restrictions and price controls. Housing prices became a critical factor driving the transformation of the real estate industry, affecting the spatial radiation effect to a certain extent, albeit less prominently^51^.

The LISA results in local areas revealed several key findings. First, there is significant potential for GPE improvement. More than half of the provinces demonstrating positive agglomeration also exhibited low–low agglomeration. Notably, the number of provinces with low–low agglomeration was more than three times that of those with high–high agglomeration, indicating that the GPE of the real estate industry in most Chinese provinces remained relatively low. Second, industrial agglomeration has emerged as an effective strategy for enhancing the GPE of the real estate sector. As the spatial radiation effect of the real estate industry becomes increasingly pronounced, the number of provinces demonstrating high–high agglomeration is rising. Finally, the influence of regional economic zones on the real estate industry needs to be leveraged. The purpose of zoning economic regions is to foster economic growth in alignment with local circumstances, necessitating increased mobility of human capital and capital resources across these zones to prevent regional developmental stagnation^52,53^.

The trend in overall spatial disparity evolution indicates several vital points. First, the surge in commodity house construction since 2010 has propelled real estate industry growth. Some regions have leveraged this expansion to boost their GPE, whereas others have experienced stagnant or declining GPE due to policy influences and construction delays. Furthermore, population migration in the twenty-first century was primarily concentrated post-2005, with the southeastward movement of the Hu Line manifesting notably after 2015^35,36^. This phenomenon has resulted in a relatively consistent east–west disparity along the Hu Line, with a recent slight increase. Additionally, compared to the population migration from west to east, the migration of technological, capital, and market factors from north to south was more pronounced^32,33,45^. Market-oriented operations within the real estate industry significantly contributed to the rapid widening of the north–south disparity.

The evolutionary trend of disparity between the eastern, central, and western regions demonstrated distinct phases. Between 2001 and 2010, the Western Development Strategy propelled rapid urbanization in the western region, fostering substantial growth in the real estate industry. Influenced by topography, transportation, and population size^54,55^, regions such as Sichuan, Chongqing, and Ningxia showed higher GPE, whereas Guizhou, Gansu, and Xinjiang experienced comparatively lower GPE. The polarization phenomenon arising from high- and low-level agglomeration was the primary driver of internal regional disparity. However, since 2011, sales of commodity houses in the eastern region have exhibited continual growth, accompanied by disparities in real estate development investments and commencement and sales areas of commodity houses across provinces. Coastal provinces, such as Fujian and Jiangsu, showed notable investment growth rates, whereas Hebei and Hainan displayed negative growth. Notably, while Beijing, Tianjin, and Jiangsu experienced significant increases in sales, Shandong and Hainan experienced significant decreases. This regional heterogeneity in investment scale and sales conditions within the real estate industry is a crucial factor contributing to the disparities within the eastern region^56^.

The evolving disparity between the northern and southern regions revealed significant trends. First, the north, predominantly characterized by heavy industry and a higher prevalence of state-owned enterprises, experienced a robust industrialization phase from 2001 to 2010. During this period, the northern region experienced rapid economic growth, surpassing that of the southern region. Consequently, house prices soared notably, particularly in regions such as the Jingjinji, Guanzhong Plain, and Central Plains Urban Regions^44^. However, as the economic center gradually shifted southward, the primary source of disparity in GPE transitioned from north to south. Second, the southern region, predominantly involved in light industry and a pivotal aspect of China's export-oriented economy, experienced accelerated economic development during the twenty-first century Maritime Silk Road initiative. This impetus led to rapid economic growth in the south, fueled by a substantial labor force and capital migration toward the region^32,33,45^. Consequently, housing prices increased significantly in the southern region. During this period, the internal disparity within the southern region began to surpass that of the northern region.

The evolving trend in the disparity between the east and west of the Hu Line delineates the contrasting policy impacts on the real estate industry before and after 2010. First, amidst the global economic crisis of 2006–2009, the State Council issued directives aimed at fostering healthy growth in the real estate market. This initiative and endeavors to construct affordable housing and renovate shanty areas significantly expanded the scale of industry investment. The increased credit availability for purchasing commodity houses indirectly boosted industry performance, leading to booming and imbalanced growth. However, since 2010, disparities in house prices have continuously widened local disparities in the real estate industry’s GPE. Notably, the emergence of green real estate has played a pivotal role in exacerbating local disparities east of the Hu Line. Additionally, supply side structural reforms, focusing on reducing excess inventory and promoting urbanization, spurred a rapid expansion of the development disparity between third- and fourth-tier cities after 2015^57^. Furthermore, population dynamics significantly contributed to the pronounced disparity east of the Hu Line, where the population density is more than twice as high as that in western areas^58^. Population concentration fosters economic development, encouraging real estate industry growth. A notable correlation exists between the development of the real estate industry and the degree of population concentration, which is indicative of space-coupling characteristics.

## Conclusions and policy recommendations

### Conclusions

Based on the triple perspectives of east–west, north–south, and east–west of the Hu Line, this study measured the GPE of China's real estate industry from 2001 to 2020 using the SDE, Moran’s index, Theil index, RKDE, and SMC to explore spatial patterns and dynamic evolution. The following primary conclusions were drawn:

1. Analyzing the spatial distribution pattern, China's real estate industry exhibits imbalanced point-surface diffusion in a static comparison. Geographically, there is an asymmetrical pattern, with higher development in the east and south and lower development in the west and north, with Henan Province as the geographical pivot. The GPE of China's real estate industry reveals positive spatial agglomeration, notably in the eastern coastal areas and middle reaches of the Yangtze River, which are marked by high–high agglomeration. Conversely, regions such as the middle reaches of the Yellow River and the northeast, southwest, and northwest regions demonstrate prevalent low–low agglomeration. Examining its dynamic evolution, the industry displayed a low GPE with a sluggish growth rate, signifying the need for further promotion of the green development model within the real estate sector. Since 2011, a noteworthy shift in the GPE geographical center of gravity has been evident, gravitating toward the southeast, indicating higher development in the south and lower development in the north. This gradual shift marks a significant characteristic of the industry's evolution, deviating from the previous tendency of higher development in the east and lower development in the west.
2. From a spatial disparity perspective, the overall national trend during the study period, notably from 2009 to 2011, followed an N-shaped development trajectory that expanded after 2011. The dichotomy between the east–west and north–south disparities is pivotal in China's real estate development. Intraregional disparities contributed to overall GPE disparity. At the local level, disparities in the scale of investments and sales within the real estate sector significantly contributed to internal disparities among the eastern, central, and western regions. The shifting economic center toward the south intensified the internal disparities between the southern and northern regions. Furthermore, population dynamics stood out as a critical factor contributing to the significant internal disparities between the eastern and western regions of the Hu Line.
3. In the context of dynamic evolutionary distribution, the convergence trend in GPE within China's real estate industry revealed a club convergence pattern. Notably, the low-, medium-, and relatively high-GPE sectors demonstrated significant convergence, whereas the high-GPE sectors exhibited weaker convergence and susceptibility to polarization. Spatial factors significantly contributed to this evolution. The continuous spatial spillover effect of GPE displayed varying impacts, with the spatial lag term exerting a weaker influence on regions with lower GPE but significantly affecting higher-level regions. The proximity to high-efficiency regions notably enhanced the efficiency of medium-level regions. Moreover, the passage of time played a crucial role in influencing the GPE mobility between regions. Time shocks exerted a minor impact on lower-level regions, but helped mitigate monopolistic tendencies in higher-level regions, enhancing overall mobility within the industry.

### Policy Recommendations

Based on the comprehensive analysis provided in the research findings, several policy recommendations are proposed to foster the green development of China's real estate industry.

1. Given the observed spatial disparities, policies should be designed to address imbalanced development across regions. Particular attention should be paid to regions exhibiting low–low agglomeration, particularly in the middle reaches of the Yellow River, as well as in the northeast, southwest, and northwest. Incentives, such as tax breaks, infrastructural investments, and subsidies, can be directed toward underdeveloped areas to stimulate growth and encourage green real estate development.
2. To facilitate the transition toward a greener real estate sector, the government should introduce policies that promote the adoption of sustainable building technologies and practices, which could include offering incentives for green building certifications, such as Leadership in Energy and Environmental Design or the Building Research Establishment Environmental Assessment Method, and financial support for the research and development of green construction materials and energy-efficient designs.
3. To mitigate the disparities between the eastern and western regions, efforts should be made to promote regional integration and cooperation, which could involve establishing cross-regional partnerships for joint infrastructure projects, sharing best practices in green development, and facilitating the exchange of skilled labor and expertise between regions.
4. Given the significant impact of population dynamics on regional disparities, population management policies should be implemented to alleviate the pressure on overpopulated areas while encouraging migration to underdeveloped regions, which could be achieved by incentivizing relocation through housing subsidies, job creation initiatives, and improved social services in less densely populated areas.

## Data Availability

The·datasets used and analyzed during the current study available from the corresponding author on reasonable request.
